# Higher non-HDL-cholesterol to HDL-cholesterol ratio linked with increased nonalcoholic steatohepatitis

**DOI:** 10.1186/s12944-018-0720-x

**Published:** 2018-04-03

**Authors:** Dianhui Wang, Ling Wang, Zhanqing Wang, Shihong Chen, Yihong Ni, Dongqing Jiang

**Affiliations:** 1 Department of Endocrinology and Metabolism, The Second Hospital of Shandong University, Jinan, 250033 Shandong China; 2 Department of Hospital-Acquired Infection Control, Laizhou City People’s Hospital, Laizhou, 261400 Shandong China; 3 Department of Emergency, Yantai Affiliated Hospital of Binzhou Medical University, Yantai, 264100 Shandong China

**Keywords:** Nonalcoholic steatohepatitis, Non-HDL-c/HDL-c ratio, Cohort study

## Abstract

**Background:**

Non-HDL-cholesterol to HDL-cholesterol (non-HDL-c/HDL-c) ratio is a feasible predictor for coronary heart disease, metabolic syndrome, and insulin resistance. Patients with nonalcoholic steatohepatitis (NASH) have an increased risk of developing cardiovascular problems and type 2 diabetes. However, the predictive role of non-HDL-c/HDL-c ratio in NASH hasn’t been investigated yet.

**Methods:**

We conducted a retrospective cohort study. A total of 3489 eligible subjects were selected in the present study. Prevalence and characteristics of NASH were demonstrated. Conditional logistic regression was used to analyze the association between non-HDL-c/HDL-c ratio and risks of NASH. Associations between non-HDL-c/HDL-c ratio and serum aminotransferase levels were also investigated.

**Results:**

The overall prevalence of NASH was 6.13%, higher in male (6.89%) than that in female (5.04%). Interestingly, the prevalence of NASH showed a positive correlation with the elevation of non-HDL-c/HDL-c ratio (Pearson’s Chi-squared test, linear trend 0.010, *p* <  0.05). The risk of NASH increased approximately 1.8-fold among subjects with higher non-HDL-c/HDL-c ratio. After adjustment for confounding factors, higher non-HDL-c/HDL-c ratio was still associated with a 54.4% increased risk of NASH. Male had higher risk of NASH than female when their non-HDL-c/HDL-c ratio increased. The risk of NASH in subjects with BMI more than 24 was 3 times higher than that in subjects with BMI less than 24. Every one unit increase in Non-HDL-c/HDL-c ratio was associated with 64.5% increase in ALT/AST level (*p* <  0.05) after adjustment for confounding factors.

**Conclusions:**

Our study provided strong evidence that subjects with higher non-HDL-c/HDL-c ratio had a higher risk of NASH, which suggested that non-HDL-c/HDL-c ratio might be a feasible predictor for NASH.

## Background

Non-alcoholic fatty liver disease (NAFLD) has emerged as the most common chronic liver disease worldwide in recent years, which was fueled by the increase in obesity and metabolic syndrome (MS) [1]. It ranges from fatty liver or hepatic steatosis to steatohepatitis with hepatic inflammation. About 5–20% of patients with fatty liver develop non-alcoholic steatohepatitis (NASH) during disease courses [2]. The morbidity and mortality in NASH patients are higher due to cardiovascular, cancer and liver-related events including hepatocellular cancer compared with the general population [3]. Importantly, the initial stages of NASH are usually symptomless, thus with the advent of NASH symptoms, liver damages have progressed to cirrhosis and become inreversible [4]. Currently liver biopsy is the only way to diagnose NASH. However, it has limitations and is ineffective in many non-advanced cases. Therefore, it is imperative to identify valuable predictors for NASH in early stages, thus to prevent further exacerbation.

Non-high-density lipoprotein cholesterol (non-HDL-c), which roughly equals to the total amount of low-density lipoprotein (LDL), very-low-density lipoprotein (VLDL), intermediate-density lipoprotein (IDL) and lipoprotein(a), is highlighted as a secondary target of lipid-lowering therapy [5]. UK Prospective Diabetes Study found that non-HDL-c/HDL-c ratio, rather than non-HDL-C, was a useful predictor for coronary heart disease (CHD) in type 2 diabetes patients [6]. Furthermore, this ratio was certified to be an effective predictor for CHD incidence in chronic kidney disease (CKD) patients [7], and an optimal predictor for MS and insulin resistance [8].

Since patients with NASH suffer an increased risk in developing cardiovascular problems, it is considerably valuable to further investigate the predictive role of non-HDL-c/HDL-c ratio in diagnosing NASH, which remains unknown. Therefore, we performed a cohort study to assess the association between the ratio of non-HDL-c to HDL-c and the risk of NASH. Our findings suggested that higher non-HDL-c/HDL-c ratio was positively associated with increased incidence of NASH. These findings highlight a pivotal role of non-HDL-c/HDL-c ratio in the diagnosis of NASH.

## Methods

### Ethics, consent and permissions

This study was approved by the ethics committee of the Second Hospital of Shandong University with written informed consents from all participants.

### Participants

Four thousand twenty-one individuals who received regular medical examination in our Hospital from 2015 to 2017 were retrospectively reviewed. Medical and surgical history for each subject was documented. The drinking histories were also recorded.

The following criteria were used for exclusion: (1) individuals with virus hepatitis; (2) alcohol abuse; (3) liver dysfunction due to cancer, autoimmune disease, genetic disease, or other disorders; (4) intake of drugs that influence serum lipids or liver function within the past 1 month; (5) hereditary hyperlipidemia. Finally, a total of 3489 eligible subjects (2061 males, 1428 females, with a mean age of 53.56 ± 11.37 years old) were selected and enrolled in the present study.

### Laboratory analyses

Blood pressure, anthropometric measurements and blood specimens were obtained by well-trained clinical staff according to a standard protocol. Blood samples were collected from all participants between 8:00 AM and 10:00 AM after a minimum 10-h fasting. All of the measurements were performed in the examination center that is affiliated to the Second Hospital of Shandong University.

Weight and height were measured in kilograms and centimeters, respectively, and BMI was calculated by dividing weight (kilograms) by the square of the height (square meters). Waist circumference was measured in centimeters. The blood pressure values were presented as the means of two measurements taken in the sitting position according to a standard protocol. The levels of alanine aminotransferase (ALT), aspartate aminotransferase (AST), fasting plasma glucose (FPG), total cholesterol (TC), triglycerides (TG), low-density lipoprotein cholesterol (LDL-c), high-density lipoprotein cholesterol (HDL-c), and Serum creatinine (Cr) were determined using an Auto Biochemical Analyzer (MODULAR-000GS; Roche, Basel, Switzerland). All participants were performed abdominal ultrasonic examination. The presence of NAFLD was detected by color ultrasonic diagnostic apparatus equipped with a 9 MHz linear-array transducer (Toshiba Aplio 500 Ultrasound Scanner).

### Diagnostic criteria and definitions

Fatty liver was diagnosed when a patient met any two of the following three ultrasonic criteria: liver and kidney echo discrepancy and presence of increased liver echogenicity (bright); unclear intrahepatic duct structure; liver far field echo decay [9]. Patients were excluded with potential cause of chronic liver disease, such as excessive alcohol consumption, hepatitis, or taking medications with a known association with fatty liver. The criterion for “nonalcoholic” is that daily alcohol consumption is lower than 20 g in women and 30 g in men. NASH is diagnosed as: 1) Serum ALT and/or AST levels were above 40 U/L; 2) the elevation was sustained greater than 6 months; 3) NAFLD [10].

Hypertension was defined as a systolic blood pressure of 140 mmHg or higher and a diastolic blood pressure of 90 mmHg or higher, or being told by a doctor or health care professional on two or more different visits that he/she had hypertension [11]. Diabetes mellitus (DM) was diagnosed if: 1) a self-reported previous diagnosis by health care professionals, 2) fasting plasma glucose 7.0 mmol/L or greater (126 mg/dL), 3) a 2-h plasma glucose in an oral glucose tolerance test 11.1 mmol/L or greater (200 mg/dL), or 4) hemoglobin A1c 6.5% or greater [12].

### Statistical analysis

Statistical analysis was performed using SPSS version 18.0 (SPSS Inc). Continuous variables were presented as mean ± SD, and categorical variables were expressed as percentages (%). Differences between groups were analyzed using one-way ANOVA for normally distributed continuous variables and chi-squared test for categorical variables. Univariate and multivariable logistic regression models were used to calculate odds ratios (OR) and 95% confidence intervals (CI) for the associations between non-HDL-c/HDL-c ratio and NASH. Two sided *p* values were calculated, and *p* <  0.05 were considered to be statistically significant.

## Results

### Baseline characteristics of the study population

Population composition and general characteristics were summarized in Table 1. Non-HDL-c/HDL-c ratios were stratified into three groups (group A: ≤ 2.13, group B: 2.13–2.89, and group C: ≥ 2.89) defined by tertiles. Significant differences were detected among the 3 groups with respect to BMI, diastolic blood pressure (DBP), TC, TG, LDLc, FPG, Cr, ALT, AST, hypertension, and DM. Compared with group A, serum levels of lipid profiles, FPG, ALT, AST, and Cr were elevated in group C. In addition, increased BMI and DBP were more likely to accompany with hypertension and DM in subjects in group C than that in group A.

**Table 1:** Baseline characteristics of the study population

Characteristic	non-HDLc/HDLc ratio			*P* value
	A (≤ 2.13)	B (2.13–2.89)	C (≥ 2.89)	
	(*n* = 1162)	(*n* = 1163)	(*n* = 1163)	
Age (yr), mean (SD)	53.26 (9.53)	53.54 (11.36)	53.79 (8.11)	0.21
BMI (kg/m^2^), mean (SD)	23.72 (3.10)	24.32 (3.20)	25.68 (3.38)	< 0.001
TC (mmol/L), mean (SD)	4.14 (1.07)	4.47 (1.04)	4.83 (1.14)	< 0.001
TG (mmol/L), median (IQR)	1.10 (1.10)	1. 46 (0.99)	1.94 (1.03)	< 0.001
LDL-C (mmol/L), mean (SD)	2.68 (0.83)	3.43 (0.76)	3.76 (0.86)	< 0.001
HDL-C (mmol/L), mean (SD)	1.40 (0.30)	1.28 (0.33)	1.11 (0.33)	0.001
FPG (mmol/L), mean (SD)	5.13 (1.72)	5.28 (1.42)	5.48 (1.68)	< 0.001
HbA1c (%), mean (SD)	5.26 (1.14)	5.31 (1.06)	6.04 (1.03)	< 0.001
SBP (mmHg), mean (SD)	124.19 (20.70)	124.74 (18.53)	125.75 (20.79)	0.36
DBP (mmHg), mean (SD)	71.51 (11.76)	75.69 (10.81)	77.75 (10.88)	< 0.001
ALT (U/L), mean (SD)	15.54 (11.42)	19.94 (10.15)	27.66 (10.79)	< 0.001
AST (U/L), mean (SD)	20.42 (8.52)	22.23 (8.24)	25.52 (9.15)	< 0.001
Cr (μmol/L), mean (SD)	70.63 (13.03)	71.99 (12.08)	73.46 (10.27)	< 0.001
DM, No. (%)	133 (11.45)	155 (13.33)	204 (17.54)	< 0.001
HT, No. (%)	468 (40.28)	489 (42.05)	526 (45.23)	0.01

Continuous variables were compared by using one-way ANOVA and categorical variables by using the Chi-squared test. P < 0.05 was considered significant

*Abbreviations*: *BMI* body mass index, *TC* total cholesterol, *TG* triglyceride, *LDL-C* low-density lipoprotein cholesterol, *HDL-C* high-density lipoprotein cholesterol, *FPG* fasting plasma glucose, *HbA1c* hemoglobin A1c, *SBP* systolic blood pressure, *DBP* diastolic blood pressure, *ALT* alanine aminotransferase, *AST* aspartate aminotrasferase, *Cr* creatinine, *DM* diabetes mellitus, *HT* hypertension

### The prevalence and distribution of NASH in the study population

**Fig. 1:**
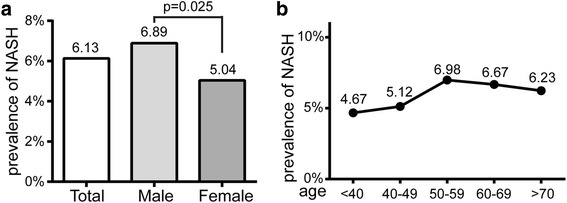
The prevalence and distribution of NASH in the study population (**a**) and according to age group (**b**)

The overall prevalence of NASH was 6.13% (Fig. 1a). Interestingly, the prevalence of NASH in male (6.89%) was higher than that in female (5.04%). The prevalence of NASH increased linearly (*p* <  0.01) with age, and reached a peak in 50–59 group, then declined slightly (Fig. 1b).

### Progressive increase of NASH with the elevation of non-HDL-c/HDL-c ratio

**Fig. 2:**
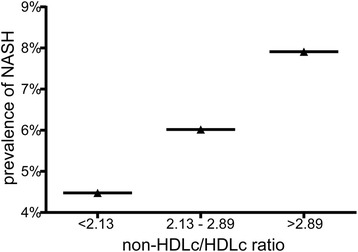
Progressive increase of NASH with the elevation of non-HDL-c/HDL-c ratio

As shown in Fig. 2, the prevalence of NASH showed positively correlation with the elevation of non-HDL-c/HDL-c ratio (Pearson’s Chi-squared test, linear trend 0.010, *p* < 0.05). As was expected, subjects in group A had the lowest prevalence of NASH. While in group C, subjects showed the highest prevalence of NASH, which was almost one time higher than that in group A.

### High risk for NASH in subjects with higher non-HDL-c/HDL-c ratio

By using conditional logistic regression analysis, we found that the risk of NASH increased approximately 1.8-fold among subjects with higher non-HDL-c/HDL-c ratio. After adjustment for confounding factors, subjects with higher non-HDL-c/HDL-c ratio were associated with 54.4% increased risk of NASH (Table 2). Compared with group A, the risk of NASH increased approximately 2.0-fold in subjects with non-HDL-c/HDL-c ratio levels greater than 2.89. These findings suggest that higher non-HDL-c/HDL-c ratio was highly positively associated with an increased risk of NASH.

**Table 2:** Conditional logistic regression analysis of non-HDLc/HDLc ratio and risk for presence of NASH

	B	SE	OR	95%CI of OR	*P* value
non-HDLc/HDLc ratio					
Univariate Model	0.456	0.095	1.778	1.309–1.982	< 0.001
Multivariate Model^a^	0.283	0.143	1.544	1.023–1.756	0.037
Group A (≤ 2.13)			1		
Group B (2.13–2.89)	0.223	0.155	1.356	1.076–1.528	< 0.001
Group C (≥ 2.89)	0.682	0.173	1.963	1.409–2.776	< 0.001

Data are coefficient (B), corresponding standard error, (SE), odds ratio (OR), 95% confidence interval (CI) and significance (*P* value)

^a^Multivariate model was adjusted for body mass index, diastolic blood pressure, total cholesterol, triglyceride, low-density lipoprotein cholesterol, high-density lipoprotein cholesterol, hemoglobin A1c, alanine aminotransferase, aspartate aminotrasferase, creatinine, presence of diabetes, hypertension

Next, we analyzed the association between non-HDL-c/HDL-c ratio and NASH after stratification by gender and BMI (Table 3). Male with increased non-HDL-c/HDL-c ratio had higher risk of NASH compared with female. Dramatically, the risk of NASH in subjects with BMI more than 24 was 3 times higher than that in subjects with BMI less than 24.

**Table 3:** Multivariate conditional logistic regression analysis of non-HDLc/HDLc ratio and risk for presence of NASH after stratification by gender and BMI

Variables	B	SE	OR	95%CI of OR	*P* value
Gender					
Male	0.716	0.151	2.047	1.522–2.753	< 0.001
Female	0.465	0.062	1.592	1.410–1.797	< 0.001
BMI					
≤ 24	0.265	0.024	1.304	1.244–1.366	< 0.001
> 24	0.874	0.175	4.316	2.137–7.523	< 0.001

Data are coefficient (B), corresponding standard error, (SE), odds ratio (OR), 95% confidence interval (CI) and significance (*P* value). Multivariate model was adjusted for diastolic blood pressure, total cholesterol, triglyceride, low-density lipoprotein cholesterol, high-density lipoprotein cholesterol, hemoglobin A1c, alanine aminotransferase, aspartate aminotrasferase, creatinine, presence of diabetes, hypertension

### Associations between non-HDL-c/HDL-c ratio and ALT/AST

Elevation of serum ALT and AST levels is a marker of abnormal liver function. Therefore, we further analyzed the associations between non-HDL-c/HDL-c ratio and serum ALT&AST levels. Compared with group A, ALT and AST levels increased approximately 2.0-fold in subjects of group C. Importantly, after adjustment for confounding factors, the associations were even more significant (Table 4). By considering it as a continuous variable, we revealed that every one unit increase in Non-HDL-c/HDL-c ratio was associated with 64.5% increase in ALT and AST levels (*p* < 0.001) after adjustment for confounding factors. These findings indicate that higher non-HDL-c/HDL-c ratio was also a predictor for the elevation of serum ALT and AST levels in NASH.

**Table 4:** Conditional logistic regression analysis of non-HDLc/HDLc ratio and serum ALT/AST levels

	B	SE	OR	95%CI of OR	*P* value
non-HDLc/HDLc ratio					
Univariate Model	0.265	0.024	1.304	1.244–1.366	< 0.001
Multivariate Model^a^	0.468	0.035	1.645	1.359–2.536	< 0.001
Group A (≤ 2.13)			1		
Group B (2.13–2.89)	0.403	0.048	1.496	1.362–1.642	< 0.001
Group C (≥ 2.89)	0.837	0.203	2.038	1.432–3.043	< 0.001

Data are coefficient (B), corresponding standard error, (SE), odds ratio (OR), 95% confidence interval (CI) and significance (*P* value)

^a^Multivariate model was adjusted for body mass index, diastolic blood pressure, total cholesterol, triglyceride, low-density lipoprotein cholesterol, high-density lipoprotein cholesterol, hemoglobin A1c, alanine aminotransferase, aspartate aminotrasferase, creatinine, presence of diabetes, hypertension

## Discussion

Our retrospective cohort study provided strong evidence for the first time that subjects with higher non-HDL-c/HDL-c ratio had a higher risk of NASH. Our study indicated that the prevalence of NASH increased progressively with the elevation of non-HDL-c/HDL-c ratio. Higher non-HDL-c/HDL-c ratio was also a predictor for the elevation of serum ALT and AST levels in NASH. Our findings expanded the predictive role of non-HDL-c/HDL-c ratio and suggested that increased non-HDL-c/HDL-c ratio might facilitate the progression of liver injury in NAFLD.

Previous studies had demonstrated the correlation between dyslipidemia and liver disease. Obesity or dyslipidemia is one of the primary risk factors of NAFLD [13]. Many clinical trials showed that statin, a lipid-lowering drug, could substantially improve liver test indexes of patients [14, 15]. The pathological process of dyslipidemia indicated that excessive cellular lipid accumulation occurred not only in adipose tissue but also in organs such as liver. Ectopic lipid accumulation in hepatocytes generated reactive oxygen species, resulting in lipid peroxidation, oxidative stress state and the releasing of several cytokines involving TNF-α, TGF-β and ILs [16]. Interestingly, Ciccone MM et al. reviewed and confirmed the role of NAFLD in increasing cardiovascular risk profile due to early impairment in vascular function and morphology [17]. The above complex inflammation circumstances led to hepatocytes apoptosis, collagen deposition, and abnormal proliferation of survived liver cells, which would further result in chronic hepatocellular injury, liver cirrhosis and eventually liver cancer [18]. Although both clinical and laboratory researches proved the correlation between dyslipidemia and liver disease, the association between non-HDL-c/HDL-c ratio and the risk of NASH haven’t been explored. Our results suggested that elevated non-HDL-c/HDL-c ratio may be a straightforward predictor for NASH and the progression of liver injury in NAFLD patients.

Serum aminotransferases such as ALT and aspartate AST can be considered as biochemical markers of liver dysfunction [19]. Elevations in ALT level are greater in patients with nonalcoholic steatohepatitis than in those with uncomplicated hepatic steatosis [20]. Moreover increased ALT level was associated with reduced insulin sensitivity, adiponectin and glucose tolerance as well as increased free fatty acids and triglycerides [21]. Elevated plasma ALT level was independently associated with increased risk of the metabolic syndrome in adults [22]. Meanwhile, elevated AST was observed in extensive tissue necrosis during myocardial infarction and in chronic liver diseases like liver tissue degeneration and necrosis [19]. AST elevations often predominated in patients with cirrhosis and even in liver diseases typically with an increased ALT [23]. The positive association with elevation of ALT and AST levels highlighted the remarkable role of non-HDL-c/HDL-c ratio in predicting chronic liver injury.

NAFLD is currently the most common chronic liver disease worldwide. By 2025 it is estimated that 25 million Americans will have NASH, of whom 20% are expected to develop cirrhosis or hepatocellular carcinoma [24]. It is expected that NASH will become the leading cause for liver transplant [25]. NAFLD/NASH is frequently described as the hepatic manifestation of metabolic syndrome [26]. As a result, patients with NASH carry a distinct set of comorbidities and risk factors that deserve special attentions [27]. Although hepatic biopsy is the gold standard to diagnose NASH, the procedure comes with a risk of major complications in 1–3% of patients and death in 0.01% of patients [28]. In this sense, noninvasively diagnostic tools was of urgent to detect the severity of steatosis or fibrosis with good accuracy [29, 30]. To get non-HDL-c/HDL-c ratio was a simple and feasible tool to predict NASH. This approach could discriminate patients with increased cardiovascular risk in NAFLD patients. However, additional studies with larger populations and gold-standard hepatic biopsy are still needed to confirm our findings.

There were several limitations of this study. Since this was a cohort study in a single center, large and multiple center studies are still needed to verify the generalizability of the results. In addition, we could not conduct a liver biopsy study to confirm the diagnosis of NAFLD/NASH. Moreover, because we used a retrospective design, the causality between non-HDL-c/HDL-c ratio and NASH cannot be fully established (i.e., we can only suggest an association). However, the strategy in this study was easily applied in large cohort study [9, 31]. A well-designed prospective research study will be necessary to address the correlations between non-HDL-c/HDL-c ratio and NASH.

## Conclusions

In summary, our results demonstrated that higher non-HDL-c/HDL-c ratio was associated with an increased risk of NASH. Our study provided a useful evidence for the primary prevention of individuals with NAFLD. Maintaining non-HDL-c/HDL-c ratio in an appropriate range, even by lifestyle interventions, might be an attractive and feasible approach to attenuate NAFLD/NASH progression.

## Abbreviations

ALT: Alanine aminotransferase
AST: Aspartate aminotransferase
Cr: Creatinine
FPG: Fasting plasma glucose
HDL-c: High-density lipoprotein cholesterol
LDL-c: Low-density lipoprotein cholesterol
NAFLD: Non-alcoholic fatty liver disease
NASH: Nonalcoholic steatohepatitis
TC: Total cholesterol
TG: Triglycerides
